# Ivan Petrovich Pavlov and Santiago Ramón y Cajal, scientists and Nobel laureates of a new century

**DOI:** 10.1590/0004-282X-ANP-2021-0460

**Published:** 2022-08-08

**Authors:** Jairo Alonso Rozo, Leonardo Palacios-Sánchez, Andrés Manuel Pérez-Acosta

**Affiliations:** 1 Fundación Universitaria los Libertadores, Integral Psychology and Human Development, Bogotá, Colombia.; 2 Universidad del Rosario, Neuroscience Group Center “Neurovitae”, Bogotá, Colombia.; 3 Universidad del Rosario, Behavioral Sciences Research Group, Bogotá, Colombia,

**Keywords:** History of Medicine, Biography, Classical Conditioning, Neurosciences, Nobel Prize, Historia de la Medicina, Biografía, Condicionamiento Clásico, Neurociencias, Premio Nobel

## Abstract

Pavlov and Cajal were two influential scientists who developed their work in the late nineteenth and early twentieth centuries. Both won the Nobel Prize in Physiology or Medicine. The authors analyze the similarities between their life and work, delving into a single aspect: the Nobel prize obtained by both with only two years of difference: Pavlov in 1904 and Cajal in 1906, shared with Camilo Golgi. Both belonged to two declining empires when nationalism was still of some importance. The theories proposed by them more than 115 years ago are still valid in much of what they contributed in their respective disciplines.

## RESUMEN

Pavlov y Cajal fueron dos científicos influyentes que desarrollaron su trabajo a finales del siglo XIX y principios del XX. Ambos ganaron el premio Nobel de Fisiología o Medicina. Los autores analizan las similitudes entre su vida y obra, profundizando en un solo aspecto: el premio Nobel obtenido por ambos con tan solo dos años de diferencia: Pavlov en 1904 y Cajal en 1906, compartido con Camilo Golgi. Ambos pertenecían a dos imperios en decadencia, en un momento en que el nacionalismo todavía tenía cierta importancia. Las teorías propuestas por ellos, hace más de 115 años, siguen vigentes en lo fundamental de lo que aportaron en sus respectivas disciplinas.

Iván Petrovich Pavlov (1849-1936) and Santiago Ramón y Cajal (1852-1934) were two major scientists who developed their work in the late 19th and early 20th centuries. Both won the Nobel Prize in Physiology or Medicine with a short chronological difference: Pavlov in 1904 and Cajal (shared with Camilo Golgi) in 1906^1^ ^-^ ^4^.

There are similarities between their life and work. Since it is impossible to cover the work of both, the authors have decided to delve into only one aspect: the Nobel Prize won by both with only two years of difference, Pavlov:1904, and Cajal:1906^5^ ^,^ ^6^.

The historical context of these two characters was marked by a strong nationalism in Europe, both in the second half of the 19^th^ century and in the first half of the 20^th^ century, which tragically unleashed the First and Second World Wars. Nationalism impacted on the scientific field, as a source of competition in achievements between countries, for example, the Nobel prizes in science: Physiology or Medicine, Physics, Chemistry, to their credit^7^.

Pavlov and Cajal both belonged to empires in decline; Russia, the home country of Pavlov, and Spain of Cajal. These two scientists, their Nobel prizes, and other distinctions obtained by them, were also of the most significant importance^8^ ^,^ ^9^.

Cajal published in 1899 the first edition of his masterpiece "Texture of the nervous system of man and vertebrates" only a year before the "Disaster of 98" occurred, caused by the war between the United States of America and Spain. After a few naval battles, all lost by Spain, the decadent empire asked to sign a peace treaty, yielding independence to Cuba, and the cession of Puerto Rico, the Philippines, and Guam to the United States, which became a colonial power^9^.

Ivan Pavlov's extensive and productive investigative career took place in Tsarist Russia and post-October 1917 Soviet Russia^8^. It was under the last two tsars, Alexander III and his son Nicholas II, that Pavlov obtained his greatest glories, such as getting the seat of the Institute of Experimental Medicine in Saint Petersburg, the honorable invitation to the conference in Madrid in 1903 (where he probably met Cajal in person), and the summit: the Nobel Prize in Medicine or Physiology, in 1904^3^. Before the fall of the Winter Palace, and although he did not agree so much with Lenin's ideals, to his fortune, revolutionary support for his prestigious investigative company was assured until he died in 1936. Thus, the famous and unstoppable Tower of Silence continued to produce scientific research beyond the radical social and economic changes that occurred due to the creation of the Soviet Union^10^.

Regarding the Nobel Prize, the juries had difficulties awarding it to Pavlov in 1904, his main competitor being Cajal^3^, who, in turn, had managed to see very precisely the "butterflies of the soul," a name he chose for the cells of the nervous system, which the German pathologist Heinrich Waldeyer (1836-1921) called neurons in 1901^11^.

The controversy also had another ingredient. Many researchers worked alone, and Pavlov was the first to research with his Ph.D. students at the St. Petersburg Institute of Experimental Medicine. Some jurors had doubts about the originality of his work^8^ ^,^ ^10^. However, once his way of working was thoroughly reviewed, they did not hesitate to designate him as the winner in 1904: "in recognition of his work in the physiology of digestion, through which knowledge on vital aspects of the matter have been transformed and increased"^5^. The speech given by Pavlov on December 12th of that year was titled: "Physiology of digestion"^4^.

Pavlov went to Stockholm to receive the award, becoming the first Russian scientist to obtain it. At 55, the scientist peaked his career: international recognition for his work and financial compensation of 73,000 gold rubles (about $ 36,000 at the time). He assigned it to his laboratory and future research^12^ ^,^ ^13^. However, curiously, Pavlov seemed not to attach much importance to such recognition. He never referred to it during the rest of his life, not even in his short autobiography. But it was an important recognition for him, his collaborators, and the nation to which he belonged^3^.

Cajal, in turn, had become the leading exponent of the "neuronal doctrine," in opposition to the "reticularists," who did not accept the existence of unicellular structures in the nervous system^14^. Camilo Golgi, who in 1880 had discovered silver staining to visualize nerve cells, was, paradoxically, a vigorous defender of the reticular theory. Cajal began to use this modified stain (double silver impregnation) in 1887. Thus, the juries also had difficulties and decided to award the prize in a shared way. The winners received the award "in recognition of their work on the structure of the nervous system." The speech delivered in French by Cajal on December 12th of that year was entitled "Structure and connections of neurons"^6^ ^,^ ^15^ ^,^ ^16^. The wise Aragonese would be the first Spaniard to receive the specific award in Physiology or Medicine^17^.

Jones (1998), cited by Grant^18^, points out that Golgi may have thought there would be a struggle between his conference and Cajal's. Having the opportunity to speak first, he misjudged the Spanish scientist's position and would have ended up attacking him. This situation would explain the controversy of his presentation. Nieto^19^ points out that Golgi was so tense that he wanted to get to the Stockholm station incognito. However, upon his arrival, a large group of people was waiting for him on the platform, including Cajal. Golgi was very nervous; he avoided any gesture of kindness with this one. According to Nieto, Golgi's attitude was because he was fully aware of the lack of updating in the bibliography on histology of the nervous system and was also concerned about a direct reply from Cajal to his speech. His state of mind is reflected in a letter from his wife, Lina Golgi, to his mother: "Camilo would run home like a runaway horse."

Finally, it would be the neural doctrine that ended up being accepted and the one that prevails, in almost all its aspects, to this day.
